# Quality of life of mothers of children and adolescents with mental health problems in Mongolia: associations with the severity of children's mental health problems and family structure

**DOI:** 10.1017/gmh.2022.34

**Published:** 2022-07-07

**Authors:** Ai Aoki, Ganchimeg Togoobaatar, Anudari Tseveenjav, Naranbaatar Nyam, Khishigsuren Zuunnast, Kenji Takehara

**Affiliations:** 1 Department of Health Policy, National Center for Child Health and Development, Setagaya, Japan; 2 Global Health Nursing, Faculty of Medicine, University of Tsukuba, Tsukuba, Ibaraki, Japan; 3 School of Nursing, Mongolian National University of Medical Sciences, Ulaanbaatar, Mongolia; 4 Department of Fundamental Nursing, Global Leadership University, Ulaanbaatar, Mongolia; 5 Department of Mental Health, School of Medicine, Mongolian National University of Medical Sciences, Ulaanbaatar, Mongolia

**Keywords:** Child and adolescent mental health, Mongolia, mothers, quality of life, WHOQOL-BREF

## Abstract

**Background:**

In low- and middle-income countries (LMICs), most parents of children with mental health problems receive limited support from social and health services while caring for their children. However, research on the quality of life (QOL) of these parents in LMICs is limited. This study aimed to investigate the association between maternal QOL and children's mental health problems, and other related factors in Mongolia.

**Methods:**

A cross-sectional analysis of children aged 4–17 years who lived in Ulaanbaatar and visited the National Mental Health Centre in Mongolia and their mothers was conducted. The mothers' QOL was assessed using the WHOQOL-BREF, and the severity of children's mental health problems was assessed using the Strengths and Difficulties Questionnaire (SDQ). Multivariate linear regression analyses were performed using the mothers' WHOQOL domain scores as dependent variables and the children's SDQ scores and demographic and socioeconomic factors as explanatory variables.

**Results:**

A total of 242 child-mother dyads were included in this study, and 231 dyads were included in the multivariate regression analyses. Children's SDQ internalising scores were negatively associated with all four maternal QOL domain scores, while their externalising scores were negatively associated with maternal physical and psychological domain scores. Non-cohabitation of fathers was negatively associated with physical, social, and environmental domain scores, and non-cohabitation of grandparents was associated with psychological and environmental domain scores.

**Conclusions:**

In Mongolia, maternal QOL is influenced by the severity of children's mental health problems and family member support. These findings highlight the importance of developing systems to support all families.

## Background

Child and adolescent mental health are increasingly important issues in low- and middle-income countries (LMICs). Globally, around 20% of children are considered to have mental health problems (Kieling *et al*., 2011), including neurodevelopmental disorders, anxiety disorders, and depression. Mental health problems in children and adolescents pose considerable burdens not only to the individuals themselves, but also to their families. Failure to address child and adolescent mental health problems is a public health issue that can eventually impede the basic development of countries in various aspects (Kieling *et al*., 2011).

Quality of life (QOL) is defined as an ‘individual's perception of their position in life in the context of the culture and value systems in which they live, and in relation to their goals, expectations, standards and concerns’ (WHO, 1996). QOL can be used to assess the status of parents of children with mental health problems. Systematic reviews of parent populations in high-income countries (HICs) have demonstrated that the QOL of parents of children with mental health problems such as autism spectrum disorder and attention-deficit/hyperactivity disorder is lower than that of parents of children without mental health problems (Vasilopoulou and Nisbet, 2016; Dey *et al*., 2019). The findings also revealed various determinants of parental QOL. Mothers experience lower QOL than fathers (Vasilopoulou and Nisbet, 2016), and the severity of children's mental health problems is associated with parental QOL (Vasilopoulou and Nisbet, 2016). Social support for parents is known to protect against stressors related to care of the children (Coyle, 2009; Vasilopoulou and Nisbet, 2016). Such social support may arise from interpersonal relationships in an individual's social network, which usually includes family members, friends, religious institutions, colleagues, or support groups (American Psychological Association, 2022). Among these forms of social support, support from family members is especially important (Alvarez *et al*., 2017).

In many LMICs, social welfare and health systems related to child and adolescent mental health are still underdeveloped (Patel *et al*., 2013). As a result, many parents of children with mental health problems in these countries face challenges, while working to make their living. They also have to cope with anxieties about their children, stigma, and social isolation. Thus, the burden on parents may be heavier in LMICs than that in HICs. However, only a few studies have investigated the QOL of parents of children with mental health problems in LMICs, and some of them have demonstrated compromised QOL in parents (Kousha *et al*., 2015; Ozgur *et al*., 2018; Karimirad *et al*., 2019). Nevertheless, while these studies have demonstrated the influence of children's mental health problems on caregivers' QOL, the determinants underlying these effects have not been elucidated.

We hypothesised that among mothers of children with mental health problems, maternal QOL correlates with the severity of the children's mental health problems and is associated with socioeconomic factors, including co-residence with family members. This study aimed to examine the association of mothers' QOL with the mental health problems of children in Mongolia.

## Methods

### Study settings

This study was conducted in Mongolia, a lower middle-income Asian country (World Bank, 2022) that is undergoing an epidemiological transition (Wahdan, 1996). Approximately 20% of children in Mongolia are suspected of having mental health problems (Aoki *et al*., 2021*a*). This study was conducted at the child and adolescent psychiatry outpatient service of the National Mental Health Centre, which is the only specialised service for child and adolescent psychiatry in Mongolia. The patients at the centre resided predominantly in Ulaanbaatar, the capital of Mongolia. However, some children visited the centre from regions outside Ulaanbaatar to seek specialised services.

### Study design

This study analysed a subsample of the population in a cross-sectional study to validate the Mongolian parental version of the Strengths and Difficulties Questionnaire (SDQ) and to demonstrate the basic characteristics of patients at the National Mental Health Centre and their families (Aoki *et al*., 2021*a*). The original study was conducted with children and their guardians who visited the National Mental Health Centre between December 2018 and March 2019. The inclusion criteria were: (1) aged <20 years; (2) written consent from parents or guardians; and (3) parents' or guardians' literacy in Mongolian. This study had no exclusion criteria.

In Mongolia, parenting is influenced by both traditional nomadic culture and the state policies during the socialist and post-socialist periods. Nomadic cultures typically consider women as the primary caregivers of family members while the modern state policies promoted socioeconomic equality between men and women (UNICEF, 2020). In the present analysis, based on considerations of differences between mothers and fathers, and between urban and rural areas, child–guardian dyads who resided in Ulaanbaatar and were accompanied by a mother were included. The children's age was restricted to 4–17 years according to the applicable age range of the SDQ.

### Measures

#### Demographic and socioeconomic factors

Information regarding participants' and their mothers' demographic and socioeconomic factors, such as household income, dwelling type, maternal educational level, maternal employment, marital status, and family structure were collected. Household income data were collected and converted into a binary variable using the threshold determined by the poverty rate among Ulaanbaatar citizens (World Bank, 2019): (1) low household income (⩽700 000 Mongolian Tugrik) and (2) middle-to-high income (>700 000 Mongolian Tugrik). Data for dwelling types were collected and converted into a binary variable: (1) apartments and (2) other dwelling types, including *gers* and simple houses. The former implies modernised living environments with basic infrastructure and the latter implies less modernised living environments with less developed infrastructure in Ulaanbaatar. Maternal education level was evaluated in terms of the highest level of educational attainment and converted into a binary variable: (1) ⩽secondary education and (2) vocational training and/or college. Maternal employment was converted into the following binary variable: (1) employed and (2) not employed. Data for family structure and co-residence of the father and grandparents (either grandmother or grandfather) were collected as binary variables: (1) cohabiting and (2) non-cohabiting.

#### Maternal QOL

The Mongolian version of the WHOQOL-BREF was used to measure maternal QOL (WHO, 1996, 1998, 2020). It is a 26-item questionnaire developed to measure health-related QOL in various cultural settings. The 26 items include 24 items related to QOL and two general questions related to overall QOL and health. Each item is scored between 1 and 5. The WHOQOL-BREF yielded scores in the physical, psychological, social relationships, and environmental domains. Each domain score ranged from 4 to 20, and these scores were evaluated as continuous variables. Although the questionnaire underwent multicultural validation during its development, it has not been validated in Mongolia (Skevington *et al*., 2004). However, a previous study utilised the Mongolian version to evaluate health-related QOL among stroke patients and their caregivers (Chuluunbaatar *et al*., 2016).

#### Severity of children's mental health problems

Severity measures of mental health problems and the primary clinical diagnoses were collected. The parental version of the SDQ was used to assess the severity of mental health problems (Goodman, 1997). The Mongolian parental version of the SDQ is the only internationally used child mental health questionnaire in Mongolia (SDQinfo, 2020), and it was validated using participants in the present study (Aoki *et al*., 2021*a*). The SDQ consists of 25 items that are scored from 0 to 2. The SDQ yields scores for emotional, peer, conduct, hyperactivity, and prosocial subscales, and each subscale score ranges from 0 to 10. Addition of the emotional and peer subscale scores yields the internalising score, and addition of the conduct and hyperactivity subscale scores yields the externalising score. Both scores range from 0 to 20. The total of the internalising and externalising scores constitutes the total difficulties score, which ranges between 0 and 40. The high-risk thresholds derived from 80 percentile scores of the normative data were 9/10 for the internalising score, 8/9 for the externalising score, and 16/17 for the total difficulties score (Aoki *et al*., 2021*a*, 2021*b*). This study used internalising and externalising scores, since a previous study demonstrated that the factor structure of the Mongolian SDQ did not fit well with the original five-subscale model (Aoki *et al*., 2021*a*). The scores were used as continuous variables, since the participants were all clinical participants, and risk classifications were not appropriate. High scores indicated higher severity of problems.

### Clinical diagnosis of children

The clinical diagnosis was based on the diagnostic criteria of the International Classification of Diseases, 10th Revision (ICD-10) (WHO, 2004). ICD-10 is conventionally used in Mongolia.

### Data collection

The SDQ and WHOQOL-BREF were completed by the guardians. Data for the other variables were collected in structured interviews conducted by trained research assistants with the participants' guardians. The participants' clinical diagnoses were retrieved from medical records.

### Analysis

Demographic and socioeconomic factors of the children and their mothers were descriptively analysed. Participants' clinical diagnosis, SDQ total difficulties score, internalising and externalising scores, and mothers' WHOQOL-BREF domain scores were descriptively analysed. The mean scores of each WHOQOL-BREF domain were compared with those obtained in a multi-country field test of the WHOQOL-BREF (Skevington *et al*., 2004). The correlations between the domain scores and general QOL and health item scores were analysed using linear regression analyses to examine their face validity.

### Multivariate linear regression analysis

The WHOQOL-BREF domain score was used as a dependent variable, while children's SDQ internalising and externalising scores, children's age, children's sex, maternal age, maternal educational level, maternal employment, household income level, dwelling type, co-residence of the father, and co-residence of grandparents were used as explanatory variables.

Correlations of the domain scores, demographic factors, and socioeconomic factors were analysed using linear regression analyses. Subsequently, multivariate linear regression analyses were performed using all explanatory variables. All explanatory variables were hypothesised to be associated with the dependent variables. Thus, a forced-entry method was used. Among independent variables, multicollinearity was assessed using a variance inflation factor. The statistical significance threshold was set at *p* = 0.05. Missing values were not imputed. Only participants without missing values for the dependent and explanatory variables were included in the analyses. All analyses were conducted using R version 3.6.2.

### Stratified analysis

To understand the difference in associations by child age and sex, stratified multivariate linear regression analyses were conducted using the same dependent variable. Children's age was categorised into adolescent (10 years old and more), and pre-adolescent (less than 10 years old) by the WHO definition (WHO, 2022). Stratifying variables were excluded from the set of explanatory variables.

### Ethical considerations

This study was approved by the ethics committees of the National Center for Child Health and Development (No. 1884) and Mongolian National University of Medical Sciences (No. 2019/D-06).

## Results

### Study participants

**Fig. 1.**
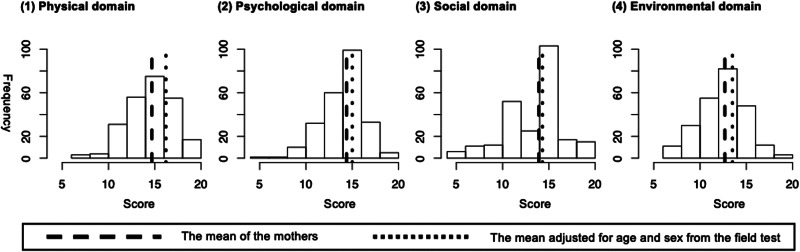
Distributions of mothers' WHOQOL-BREF domain scores and comparisons with the multi-country field test results.

Among the 498 child–guardian dyads of the original study, 242 child–mother dyads were included in the present analysis. The mean age of the children was 10.3 years [standard deviation (s.d.), 3.7 years], and 142 (58.7%) children were male. The mean maternal age was 38.1 years (s.d., 6.7 years). Data describing the demographic and socioeconomic factors are summarised in Table 1. Regarding the children's clinical diagnoses, the most common ICD-10 category was F7 intellectual disability (29.1%), followed by F9 behavioural and emotional disorders with onset usually occurring in childhood and adolescence (20.7%), F8 pervasive and specific developmental disorders (19.4%), and F4 anxiety, dissociative, stress-related, somatoform, and other non-psychotic mental disorders (15.4%). The mean SDQ total difficulties score was 20.4 (s.d., 6.4, 70.5% above the high-risk threshold). The mean internalising and externalising scores were 10.4 (s.d., 3.5, 57.3% above the high-risk threshold) and 10.0 (s.d., 4.2, 64.5% above the high-risk threshold), respectively. The mean scores for the WHOQOL-BREF physical, psychological, social, and environmental domains were 14.7 (s.d., 2.4), 14.4 (s.d., 2.2), 13.9 (s.d., 3.3), and 12.7 (s.d., 2.6), respectively. The item-based summary is presented in online Supplementary Table S1. All mean values were lower than the age- and sex-adjusted mean values from the field test (Fig. 1) (Skevington *et al*., 2004). All domain scores were significantly correlated with general QOL and general health scores. The psychological and environmental domain scores were strongly associated with general QOL scores. The physical and psychological domain scores were strongly associated with general health scores (Table 2).

**Table 1.** Demographic and socioeconomic characteristics of the children and mothers

	*N*	%
Children's age, mean (s.d.)	10.3 (3.7)	
Children's sex		
Male	142	58.7
Female	100	41.3
Mothers' age, mean (s.d.)	38.1 (6.7)	
Maternal educational levels		
>Secondary education	138	57.3
**⩽**Secondary education	103	42.7
Maternal employment		
Employed	226	93.8
Not employed	15	6.2
Marital status		
Married	183	76.6
Not married	56	23.4
Household income levels		
Middle-high income	163	67.9
Low income	77	32.1
Dwelling types		
Apartment	111	45.9
Others (including Ger)	131	54.1
Co-residence of the father		
Yes	179	74.6
No	61	25.4
Co-residence of grandparents		
Yes	50	20.8
No	190	79.2

**Table 2.** Summary of mothers' WHOQOL-BREF, associations between domain scores and general item scores, and comparisons with field test results

WHOQOL-BREF	Mean (s.d.)	Correlation with general QOL score Coefficient (95% CI)	Correlation with general health score Coefficient (95% CI)
Physical domain	14.7 (2.4)	0.15 (0.11–0.19)	0.23 (0.19–0.27)
Psychological domain	14.4 (2.2)	0.21 (0.18–0.25)	0.20 (0.16–0.25)
Social domain	13.9 (3.3)	0.12 (0.09–0.15)	0.11 (0.08–0.14)
Environmental domain	12.7 (2.6)	0.18 (0.14–0.21)	0.13 (0.09–0.17)

### Relationships between QOL in mothers and the severity of children's mental health problems and other socioeconomic factors

Among the 242 child–mother dyads, 231 dyads without missing values for the independent and dependent variables were included in the regression analyses. Univariate linear regression analyses demonstrated significant associations of all four WHOQOL-BREF domain scores with the SDQ internalising score, maternal education level, household income level, dwelling type, and co-residence of the father. Significant associations were found between a part of the domain scores and the SDQ externalising score, maternal age, and co-residence of grandparents (online Supplementary Table S2).

In the multivariate linear regression analyses, each WHOQOL-BREF domain score was used as the dependent variable. The physical domain score was significantly associated with the SDQ internalising score [adjusted coefficient, −0.12 (−0.21 to −0.03), *p* = 0.008], the SDQ externalising score [adjusted coefficient, −0.10 (−0.17 to −0.02), *p* = 0.02], maternal age [adjusted coefficient, −0.07 (−0.12 to −0.02), *p* = 0.004], and non-cohabitation with the father [adjusted coefficient, −0.74 (−1.46 to −0.01), *p* = 0.048]. The psychological domain score was significantly associated with the SDQ internalising score [adjusted coefficient, −0.14 (−0.23 to −0.06), *p* = 0.001], SDQ externalising score [adjusted coefficient, −0.08 (−0.15 to −0.01), *p* = 0.003], and non-cohabitation with grandparents [adjusted coefficient, −0.81 (−1.49 to −0.14), *p* = 0.02]. The social domain score was significantly associated with the SDQ internalising score [adjusted coefficient, −0.18 (−0.30 to −0.06), *p* = 0.004] and non-cohabitation with the father [adjusted coefficient, −2.07 (−3.05 to −1.09), *p* < 0.001]. Mothers' unemployment and non-cohabitation with grandparents demonstrated marginal associations. The environmental domain score was significantly associated with the SDQ internalising score [adjusted coefficient, −0.13 (−0.23 to −0.04), *p* = 0.006], dwelling types other than apartment [adjusted coefficient, −0.71 (−1.40 to −0.01), *p* = 0.047], non-cohabitation with the father [adjusted coefficient, −1.06 (−1.82 to −0.30), *p* = 0.007], and non-cohabitation with grandparents [adjusted coefficient, −1.16 (−1.91 to −0.40), *p* = 0.003]. These results are presented in Table 3. The variance inflation factors were below 2 for all explanatory variables. Stratified analyses demonstrated the similar overall associations between mothers' QOL, children's SDQ scores, socioeconomic factors, and family structure. In addition to the associations demonstrated in the main multivariate analyses, age-stratified analyses demonstrated that the physical domain score was significantly associated with maternal education among young children, and the social domain score was significantly associated with maternal unemployment and low household income. Sex-stratified analyses demonstrated that dwelling types other than apartment were significantly associated with the physical domain score among participants with male children, and the physical domain score was associated with non-cohabitation with grandparents, the social domain score with dwelling types other than apartment and non-cohabitation with grandparents, and the environmental domain score with the SDQ externalisation score among participants with female children. The results of the stratified analyses are presented in online Supplementary Tables S3 and S4.

**Table 3.** Multiple regression analyses using the WHOQOL-BREF domain scores as dependent variables

	Physical domain		Psychological domain		Social domain		Environmental domain	
	*β* coefficients (95% CI)	*p* value	*β* coefficients (95% CI)	*p* value	*β* coefficients (95% CI)	*p* value	*β* coefficients (95% CI)	*p* value
SDQ internalising score	−0.12 (−0.21 to −0.03)	0.008	−0.14 (−0.23 to −0.06)	0.001	−0.18 (−0.30 to −0.06)	0.004	−0.13 (−0.23 to −0.04)	0.006
SDQ externalising score	−0.10 (−0.17 to −0.02)	0.015	−0.08 (−0.15 to −0.01)	0.03	0.02 (−0.09 to 0.12)	0.77	−0.04 (−0.12 to 0.04)	0.28
Child age	0.02 (−0.07 to 0.11)	0.61	−0.02 (−0.10 to 0.06)	0.63	−0.03 (−0.14 to 0.09)	0.67	−0.01 (−0.11 to 0.08)	0.78
Child sex (female *v.* male^a^)	−0.03 (−0.62 to 0.57)	0.93	−0.09 (−0.64 to 0.47)	0.76	−0.29 (−1.09 to 0.51)	0.47	−0.35 (−0.97 to 0.27)	0.27
Maternal age	−0.07 (−0.12 to −0.02)	0.004	−0.01 (−0.06 to 0.03)	0.57	−0.02 (−0.08 to 0.05)	0.59	0 (−0.05 to 0.05)	0.86
Maternal education level (middle/low *v.* high^a^)	−0.27 (−0.96 to 0.43)	0.45	−0.36 (−1.02 to 0.29)	0.28	−0.02 (−0.96 to 0.92)	0.97	−0.11 (−0.84 to 0.62)	0.78
Maternal employment (unemployed *v.* employed^a^)	0.06 (−1.12 to 1.23)	0.93	−0.57 (−1.67 to 0.53)	0.31	−1.48 (−3.06 to 0.09)	0.07	0.15 (−1.07 to 1.38)	0.81
Household income level (low *v.* middle/high^a^)	−0.38 (−1.12 to 0.37)	0.32	−0.25 (−0.94 to 0.45)	0.49	−0.89 (−1.90 to 0.11)	0.08	−0.53 (−1.31 to 0.25)	0.18
Dwelling type (others *v.* apartments^a^)	−0.64 (−1.31 to 0.03)	0.06	−0.21 (−0.84 to 0.41)	0.50	−0.42 (−1.32 to 0.47)	0.35	−0.71 (−1.40 to −0.01)	0.047
Father (not cohabiting *v.* cohabiting^a^)	−0.74 (−1.46 to −0.01)	0.048	−0.62 (−1.30 to 0.06)	0.07	−2.07 (−3.05 to −1.09)	<0.001	−1.06 (−1.82 to −0.30)	0.007
Grandparents (not cohabiting *v.* cohabiting^a^)	−0.55 (−1.27 to 0.18)	0.14	−0.81 (−1.49 to −0.14)	0.02	−0.93 (−1.91 to 0.04)	0.06	−1.16 (−1.91 to −0.40)	0.003

a Indicate the reference categories.

## Discussion

This study investigated the association of various aspects of the QOL of mothers with the severity of the children's mental health problems and other socioeconomic factors. All four QOL domains of mothers showed significant associations with the severity of the children's mental health problems. Children's internalising problems affected more QOL domains than externalising problems. When marginal-level associations were also considered, co-residence with fathers and grandparents was associated with better QOL in most domains for mothers. In addition, a higher maternal age was associated with a poorer physical QOL and dwelling types other than apartments with a poorer environmental QOL.

The results suggest that the QOL of mothers of children with mental health problems may be lower than that of healthy adults, although the present study did not collect data from comparatively healthy adults. All four domain scores were lower than those in the multi-country field test (Skevington *et al*., 2004). In comparison with the WHOQOL-BREF domain scores of caregivers of post-stroke patients in Mongolia, those of mothers of children with mental health problems were lower for the psychological, social, and environmental domains and higher for the physical domain (Chuluunbaatar *et al*., 2016). To make the results comparable with the stroke patient caregiver study, and adjusted scale of 0–100 was used (WHO, 1998). These comparisons suggest that mothers of children with mental health problems have a poorer QOL that is comparable to that of caregivers of people with highly disabling physical diseases (Chuluunbaatar *et al*., 2016). The results, together with evidence from HICs and some LMICs, imply that poor QOL in mothers of children with mental health problems is a universal problem (Vasilopoulou and Nisbet, 2016; Dey *et al*., 2019). In this study, the mean environmental domain scores were the lowest among the four domains. The scores for two items on financial resources, and recreation and leisure, were poor and contributed to poor environmental QOL (online Supplementary Table S1). Even though the Mongolian government provides financial supports for families, such as disability pensions, to respond to this need, further investigation to improve the well-being of children and their parents should be undertaken.

Our study demonstrated a significant association between maternal QOL and the severity of mental health problems in children. This is consistent with the findings of a previous systematic review (Vasilopoulou and Nisbet, 2016). However, maternal mental health problems are also known to influence child mental health problems (Leis *et al*., 2014). Considering these reciprocal relationships, supporting parents and addressing poor QOL in mothers of children with mental health problems will eventually benefit their children's mental health.

Our study also demonstrated the protective roles of fathers and grandparents in the mothers' QOL. In our study, 74.6% and 20.8% of mothers lived with the father of the child and the grandparents, which is associated with better QOL in mothers. Single-parent families are increasing and household sizes are decreasing globally (United Nations Department of Economic and Social Affairs, 2017). In Mongolia, the higher life stress of single mothers has been documented previously. Single mothers are reported to suffer from both financial risk and social withdrawal (World Bank, 2018). Also, there is a unique type of single-mother household where mothers move to urban area with their children to complete their basic education and fathers live separately to continue their business such as animal husbandry. This resulted in a double-burden on women in Mongolia (UNICEF, 2020).

On the contrary, the key variables concerning socioeconomic positions such as maternal education, maternal employment, and household income demonstrated significant association by the univariate analyses and by a part of the age-stratified analyses and failed to demonstrate significant association by the main analyses. Although there was no multicollinearity, these socioeconomic factors are assumed to interact with each other to some extent. Hence, they failed to be statistically significant in the multivariate analyses. Mongolian social backgrounds may actually minimise the impact of these factors. For example, the school enrolment rate has been remarkably high (World Bank, 2022). Among study participants, 92% of the mothers completed secondary education. Above secondary education, the length of education may not influence their QOL significantly. Also, the maternal employment rate is high. Among the participants, 94% were employed. This made it difficult to show the impact of maternal employment such as getting social support from work places and getting extra burden to daily lives. In addition, household income was not significantly associated with maternal QOL. The basic social services such as education and healthcare are accessible regardless of income in Mongolia, which may result in a smaller QOL gap by economic status. Also, there is a possibility that monetary income might not reflect the economic status appropriately in Mongolian society. The results of the stratified analyses imply that there is cultural difference in child-rearing by child sex, and the increasing need for economic stability and wider social network as children get older. Further research is necessary to identify the needs of families of children with mental health problems and formulate policies to better support them.

Establishment of support systems for parents with low familial support is an urgent issue. Furthermore, considering the scarcity of professional resources to support children with mental health problems, parent-mediating programmes for neurodevelopmental disorders that directly involve parents as trainers in managing their children's difficulties are gaining attention in some LMICs (Koly *et al*., 2021). The World Health Organization has developed a caregiver skills training programme for families with children with developmental difficulties or delays and has been trying to promote the programme (Salomone *et al*., 2019). The involvement of parents in these programmes improves the children's mental health problems, increases parents' knowledge and parenting skills, and improves their QOL (Coren *et al*., 2018; Musetti *et al*., 2021). In Uganda, a community-based, peer-led parenting programme has been shown to positively influence both children's development and maternal mental health (Singla *et al*., 2015). Despite the limited resources for developing mental health systems in LMICs, the focus should not be solely on diagnosing or providing treatments for children with mental health problems but should also include supporting the entire families rearing these children.

## Limitations

This study had some limitations. First, the Mongolian version of the WHOQOL was not validated in Mongolia, nor did the present study recruit a comparison group of mothers of children without mental health problems. However, since the questionnaire was developed for use in various cultural settings and each WHOQOL domain score demonstrated a correlation with general QOL and health items, the WHOQOL-BREF is considered to be the most appropriate QOL measure available in Mongolia.

Second, the children's clinical diagnoses were diverse. However, this study aimed to evaluate the association between maternal QOL and the severity of mental health problems in children with socioeconomic factors, and to demonstrate the need to support the entire family. Thus, recruitment of a clinically heterogeneous population of children was considered acceptable. Third, it is possible that there was low representativeness of the study population to Mongolian children with mental health problems. Similar to the LMICs, awareness of mental health problems and utilisation of psychiatric services is low in Mongolia. A considerably low proportion of the children seek care and the National Mental Health Centre is the only tertiary level of specialised child and adolescent psychiatric service in the country. In addition, our study findings may not generalisable to the rural area where the majority of the households live as extended family. Fourth, this study did not diagnose mental health problems of the mothers. Although the questionnaire included a question about their current mental health problems, less than 5% of participants answered that they have mental health problems. Most answers were not specified and some were general expressions such as depressive mood and stress. Awareness of mental health problems is not high in Mongolia and people rarely seek professional help. Thus, this was not included in the explanatory variables in this analysis.

## Conclusions

The QOL of mothers of children with mental health problems is significantly influenced by the severity of their children's mental health problems and is protected by cohabiting fathers and grandparents. Thus, to develop child and adolescent mental health systems in LMICs, it is important to support the entire families who care for children with mental health problems, even though available resources are limited.

## Supplementary material

For supplementary material accompanying this paper visit http://dx.doi.org/10.1017/gmh.2022.34. click here to view supplementary material
