# Supplementary material for: Quality of life of mothers of children and adolescents with mental health problems in Mongolia: associations with the severity of children's mental health problems and family structure
Source: Glob Ment Health (Camb). 2022 Jul 7;9:298–305. doi: 10.1017/gmh.2022.34 (PMC9806993; doi:10.1017/gmh.2022.34)
Supplement: Supplementary file 1 [file S2054425122000346sup.zip › S2054425122000346sup001.docx]

**Supplementary Table 1. The Summary of the WHOQOL-BREF**

| Items | 1 (Poor QOL) | 2 | 3 | 4 | 5 (Good QOL) | Mean |
| --- | --- | --- | --- | --- | --- | --- |
| General QOL | 5 (2.1%) | 21 (8.8%) | 86 (36%) | 111 (46.4%) | 16 (6.7%) | 3.47 |
| General health | 4 (1.7%) | 40 (16.8%) | 78 (32.8%) | 101 (42.4%) | 15 (6.3%) | 3.35 |
| Physical domain | |  |  |  |  |  |
| Pain and discomfort | 1 (0.4%) | 20 (8.3%) | 66 (27.4%) | 63 (26.1%) | 91 (37.8%) | 3.93 |
| Dependence on medicinal substances and medical aids | 3 (1.3%) | 40 (16.7%) | 51 (21.3%) | 53 (22.2%) | 92 (38.5%) | 3.80 |
| Energy and fatigue | 6 (2.5%) | 20 (8.3%) | 81 (33.8%) | 114 (47.5%) | 19 (7.9%) | 3.50 |
| Mobility | 4 (1.7%) | 7 (2.9%) | 23 (9.5%) | 118 (49%) | 89 (36.9%) | 4.17 |
| Sleep and rest | 16 (6.6%) | 56 (23.2%) | 62 (25.7%) | 89 (36.9%) | 18 (7.5%) | 3.15 |
| Activities of daily living | 8 (3.3%) | 20 (8.3%) | 69 (28.6%) | 123 (51%) | 21 (8.7%) | 3.54 |
| Working capacity | 5 (2.1%) | 19 (7.9%) | 64 (26.8%) | 126 (52.7%) | 25 (10.5%) | 3.62 |
| Psychological domain | |  |  |  |  |  |
| Positive feelings | 4 (1.7%) | 25 (10.4%) | 73 (30.4%) | 123 (51.3%) | 15 (6.3%) | 3.50 |
| Spirituality, religion and personal beliefs | 3 (1.2%) | 9 (3.7%) | 51 (21.2%) | 148 (61.4%) | 30 (12.4%) | 3.80 |
| Thinking learning, memory and concentration | 1 (0.4%) | 13 (5.4%) | 66 (27.6%) | 135 (56.5%) | 24 (10%) | 3.70 |
| Body image and appearance | 10 (4.1%) | 15 (6.2%) | 53 (22%) | 115 (47.7%) | 48 (19.9%) | 3.73 |
| Self-esteem | 3 (1.3%) | 13 (5.5%) | 65 (27.4%) | 126 (53.2%) | 30 (12.7%) | 3.71 |
| Negative feelings | 12 (5%) | 48 (20.1%) | 87 (36.4%) | 80 (33.5%) | 12 (5%) | 3.13 |
| Social domain | |  |  |  |  |  |
| Personal relationships | 7 (2.9%) | 18 (7.5%) | 44 (18.3%) | 145 (60.2%) | 27 (11.2%) | 3.69 |
| Sexual activity | 17 (7.2%) | 23 (9.8%) | 53 (22.6%) | 121 (51.5%) | 21 (8.9%) | 3.45 |
| Social support | 25 (10.5%) | 29 (12.2%) | 54 (22.7%) | 108 (45.4%) | 22 (9.2%) | 3.31 |
| Environmental domain | |  |  |  |  |  |
| Freedom, physical safety and security | 9 (3.8%) | 24 (10%) | 80 (33.3%) | 106 (44.2%) | 21 (8.8%) | 3.44 |
| Physical environment | 18 (7.5%) | 39 (16.2%) | 85 (35.3%) | 85 (35.3%) | 14 (5.8%) | 3.16 |
| Financial resources | 37 (15.4%) | 48 (20%) | 85 (35.4%) | 54 (22.5%) | 16 (6.7%) | 2.85 |
| Opportunities for acquiring new information and skills | 12 (5%) | 35 (14.6%) | 88 (36.8%) | 82 (34.3%) | 22 (9.2%) | 3.28 |
| Participation in and opportunities for recreation and leisure activities | 36 (15%) | 69 (28.8%) | 85 (35.4%) | 38 (15.8%) | 12 (5%) | 2.67 |
| Home environment | 7 (2.9%) | 36 (14.9%) | 63 (26.1%) | 108 (44.8%) | 27 (11.2%) | 3.47 |
| Health and social care: accessibility and quality | 20 (8.4%) | 28 (11.7%) | 92 (38.5%) | 86 (36%) | 13 (5.4%) | 3.18 |
| Transport | 21 (8.8%) | 29 (12.1%) | 72 (30%) | 94 (39.2%) | 24 (10%) | 3.30 |
