# Supplementary material for: Quality of life of mothers of children and adolescents with mental health problems in Mongolia: associations with the severity of children's mental health problems and family structure
Source: Glob Ment Health (Camb). 2022 Jul 7;9:298–305. doi: 10.1017/gmh.2022.34 (PMC9806993; doi:10.1017/gmh.2022.34)
Supplement: Supplementary file 1 [file S2054425122000346sup.zip › S2054425122000346sup002.docx]

**Supplementary Table 2. Univariate linear regression analyses**

|  | Physical domain | | Psychological domain | | Social domain | | Environmental domain | |
| --- | --- | --- | --- | --- | --- | --- | --- | --- |
|  | β coefficients (95% CI) | p value | β coefficients (95% CI) | p value | β coefficients (95% CI) | p value | β coefficients (95% CI) | p value |
| SDQ internalizing score | -0.21 (-0.30, -0.13) | < 0.001 | -0.22 (-0.29, -0.14) | < 0.001 | -0.26 (-0.37, -0.14) | < 0.001 | -0.21 (-0.30, -0.13) | < 0.001 |
| SDQ externalizing score | -0.16 (-0.23, -0.09) | < 0.001 | -0.15 (-0.22, -0.08) | < 0.001 | -0.08 (-0.18, 0.02) | 0.11 | -0.13 (-0.20, -0.05) | 0.001 |
| Child age | -0.04 (-0.12, 0.04) | 0.36 | -0.04 (-0.11, 0.04) | 0.35 | -0.09 (-0.20, 0.02) | 0.11 | -0.04 (-0.13, 0.05) | 0.37 |
| Child sex (female vs. male*) | -0.15 (-0.79, 0.49) | 0.64 | -0.23 (-0.81, 0.35) | 0.44 | -0.36 (-1.21, 0.49) | 0.41 | -0.48 (-1.14, 0.17) | 0.15 |
| Maternal age | -0.07 (-0.11, -0.02) | 0.004 | -0.03 (-0.07, 0.02) | 0.22 | -0.05 (-0.12, 0.01) | 0.09 | -0.02 (-0.07, 0.03) | 0.38 |
| Maternal education level (middle/low vs. high*) | -1.28 (-1.89, -0.67) | < 0.001 | -1.11 (-1.68, -0.55) | < 0.001 | -1.31 (-2.14, -0.48) | 0.002 | -1.23 (-1.86, -0.6) | < 0.001 |
| Maternal employment (unemployed vs. employed*) | -0.34 (-1.65, 0.97) | 0.61 | -0.77 (-1.97, 0.42) | 0.21 | -1.69 (-3.43, 0.05) | 0.06 | -0.06 (-1.42, 1.29) | 0.93 |
| Household Income level (low vs. middle/high*) | -1.49 (-2.13, -0.85) | < 0.001 | -1.18 (-1.77, -0.59) | < 0.001 | -2.31 (-3.15, -1.46) | < 0.001 | -1.63 (-2.29, -0.98) | < 0.001 |
| Dwelling type (others vs. apartments*) | -1.28 (-1.88, -0.67) | < 0.001 | -0.89 (-1.45, -0.32) | 0.002 | -1.24 (-2.06, -0.41) | 0.003 | -1.32 (-1.95, -0.7) | < 0.001 |
| Father (not cohabiting vs. cohabiting*) | -1.31 (-2.01, -0.60) | < 0.001 | -1.01 (-1.66, -0.35) | 0.003 | -2.71 (-3.61, -1.80) | < 0.001 | -1.49 (-2.21, -0.76) | < 0.001 |
| Grandparents (not cohabiting vs. cohabiting*) | -0.58 (-1.35, 0.20) | 0.14 | -0.75 (-1.45, -0.04) | 0.04 | -0.6 (-1.63, 0.44) | 0.26 | -0.95 (-1.74, -0.16) | 0.02 |

*Asterisks indicate the reference categories.
