# Supplementary material for: Quality of life of mothers of children and adolescents with mental health problems in Mongolia: associations with the severity of children's mental health problems and family structure
Source: Glob Ment Health (Camb). 2022 Jul 7;9:298–305. doi: 10.1017/gmh.2022.34 (PMC9806993; doi:10.1017/gmh.2022.34)
Supplement: Supplementary file 1 [file S2054425122000346sup.zip › S2054425122000346sup003.docx]

**Supplementary table 3. Multiple regression analyses stratified by child age using the WHOQOL-BREF domain scores as dependent variables**

| Children aged younger than 10 years old (n=113) | Physical domain | | Psychological domain | | Social domain | | Environmental domain | |
| --- | --- | --- | --- | --- | --- | --- | --- | --- |
|  | Estimate (95%CI) | p-value | Estimate (95%CI) | p-value | Estimate (95%CI) | p-value | Estimate (95%CI) | p-value |
| SDQ internalising score | -0.04 (-0.18,0.09) | 0.56 | -0.04 (-0.18,0.10) | 0.56 | -0.02 (-0.21,0.16) | 0.79 | -0.07 (-0.21,0.07) | 0.35 |
| SDQ externalising score | -0.08 (-0.19,0.03) | 0.15 | -0.09 (-0.20,0.02) | 0.11 | 0.03 (-0.13,0.18) | 0.74 | -0.02 (-0.14,0.09) | 0.70 |
| Child sex (female vs male) | 0.22 (-0.65,1.09) | 0.61 | -0.18 (-1.05,0.70) | 0.69 | 0 (-1.20,1.21) | 1.00 | -0.29 (-1.19,0.62) | 0.53 |
| Maternal age | -0.05 (-0.11,0.01) | 0.12 | -0.02 (-0.08,0.04) | 0.50 | 0 (-0.08,0.08) | 0.98 | 0 (-0.06,0.07) | 0.89 |
| Maternal education levels (middle/low vs high*) | -1.28 (-2.28,-0.28) | 0.01 | -0.75 (-1.76,0.26) | 0.14 | -1.28 (-2.67,0.10) | 0.07 | -0.61 (-1.65,0.43) | 0.25 |
| Maternal employment (unemployed vs employed*) | 1.32 (-0.25,2.89) | 0.10 | 0.29 (-1.30,1.87) | 0.72 | 0.81 (-1.36,2.98) | 0.46 | 0.86 (-0.77,2.50) | 0.30 |
| Household income level (low vs middle/high*) | 0.23 (-0.84,1.3) | 0.67 | 0.16 (-0.92,1.24) | 0.77 | 0.16 (-1.32,1.64) | 0.83 | -0.35 (-1.46,0.77) | 0.54 |
| Dwelling type (others vs apartments*) | -0.87 (-1.78,0.05) | 0.06 | -0.72 (-1.64,0.21) | 0.13 | -0.33 (-1.60,0.93) | 0.60 | -0.53 (-1.49,0.43) | 0.27 |
| Father (not cohabiting, cohabiting*) | -0.79 (-1.86,0.27) | 0.14 | -0.75 (-1.82,0.32) | 0.17 | -2.81 (-4.28,-1.34) | <0.001 | -1.81 (-2.92,-0.70) | 0.002 |
| Grandparents (not cohabiting, cohabiting*) | -0.89 (-1.85,0.06) | 0.07 | -1.14 (-2.10,-0.18) | 0.02 | -1.11 (-2.43,0.21) | 0.10 | -1.25 (-2.25,-0.26) | 0.01 |
| Children aged 10 years and older (n=118) | Physical domain | | Psychological domain | | Social domain | | Environmental domain | |
|  | Estimate (95%CI) | p value | Estimate (95%CI) | p value | Estimate (95%CI) | p value | Estimate (95%CI) | p value |
| SDQ internalising score | -0.16 (-0.28,-0.03) | 0.02 | -0.19 (-0.31,-0.08) | 0.001 | -0.27 (-0.44,-0.11) | 0.001 | -0.17 (-0.30,-0.03) | 0.02 |
| SDQ externalising score | -0.12 (-0.23,-0.01) | 0.03 | -0.06 (-0.15,0.04) | 0.23 | -0.01 (-0.15,0.13) | 0.90 | -0.09 (-0.20,0.03) | 0.14 |
| Child sex (female vs male*) | -0.04 (-0.89,0.80) | 0.92 | 0.09 (-0.65,0.84) | 0.80 | -0.25 (-1.33,0.83) | 0.65 | -0.31 (-1.21,0.59) | 0.50 |
| Maternal age | -0.07 (-0.15,0) | 0.045 | 0 (-0.07,0.06) | 0.91 | -0.02 (-0.12,0.07) | 0.61 | 0 (-0.08,0.07) | 0.93 |
| Maternal education levels (middle/low vs high*) | 0.52 (-0.47,1.51) | 0.30 | -0.12 (-1.00,0.77) | 0.79 | 0.88 (-0.39,2.16) | 0.17 | 0.28 (-0.79,1.34) | 0.61 |
| Maternal employment (unemployed vs employed*) | -1.15 (-2.92,0.63) | 0.20 | -1.56 (-3.14,0.02) | 0.053 | -3.79 (-6.07,-1.52) | 0.001 | -0.7 (-2.60,1.21) | 0.47 |
| Household income level (low vs middle/high*) | -0.82 (-1.89,0.26) | 0.13 | -0.61 (-1.56,0.35) | 0.21 | -1.51 (-2.89,-0.14) | 0.03 | -0.49 (-1.65,0.66) | 0.40 |
| Dwelling type (others vs apartments*) | -0.56 (-1.55,0.43) | 0.26 | 0.07 (-0.81,0.95) | 0.88 | -0.78 (-2.05,0.48) | 0.22 | -1.01 (-2.07,0.05) | 0.06 |
| Father (not cohabiting, cohabiting*) | -0.70 (-1.72,0.33) | 0.18 | -0.61 (-1.52,0.30) | 0.19 | -1.47 (-2.78,-0.16) | 0.03 | -0.43 (-1.52,0.67) | 0.44 |
| Grandparents (not cohabiting, cohabiting*) | -0.18 (-1.30,0.93) | 0.75 | -0.48 (-1.48,0.51) | 0.34 | -0.76 (-2.19,0.66) | 0.29 | -1.25 (-2.44,-0.05) | 0.04 |

* Asterisks indicate the reference categories.
