# Supplementary material for: Quality of life of mothers of children and adolescents with mental health problems in Mongolia: associations with the severity of children's mental health problems and family structure
Source: Glob Ment Health (Camb). 2022 Jul 7;9:298–305. doi: 10.1017/gmh.2022.34 (PMC9806993; doi:10.1017/gmh.2022.34)
Supplement: Supplementary file 1 [file S2054425122000346sup.zip › S2054425122000346sup004.docx]

**Supplementary table 4. Multiple regression analyses stratified by child sex using the WHOQOL-BREF domain scores as dependent variables**

| Male participants (n=137) | Physical domain | | Psychological domain | | Social domain | | Environmental domain | |
| --- | --- | --- | --- | --- | --- | --- | --- | --- |
|  | Estimate (95%CI) | p value | Estimate (95%CI) | p value | Estimate (95%CI) | p value | Estimate (95%CI) | p value |
| SDQ internalising score | -0.12 (-0.23,-0.01) | 0.03 | -0.16 (-0.26,-0.06) | 0.001 | -0.19 (-0.35,-0.02) | 0.02 | -0.08 (-0.2,0.03) | 0.15 |
| SDQ externalising score | -0.12 (-0.21,-0.02) | 0.02 | -0.09 (-0.19,0) | 0.04 | 0.03 (-0.12,0.18) | 0.70 | 0.03 (-0.07,0.14) | 0.53 |
| Child age | 0.01 (-0.10,0.12) | 0.87 | -0.01 (-0.11,0.09) | 0.84 | 0 (-0.16,0.16) | 0.99 | -0.02 (-0.14,0.10) | 0.74 |
| Maternal age | -0.08 (-0.14,-0.02) | 0.01 | -0.02 (-0.08,0.03) | 0.40 | -0.05 (-0.14,0.04) | 0.28 | -0.01 (-0.07,0.05) | 0.74 |
| Maternal education levels (middle/low vs high*) | -0.13 (-1.07,0.82) | 0.79 | -0.54 (-1.40,0.33) | 0.22 | -0.46 (-1.87,0.94) | 0.51 | -0.13 (-1.14,0.87) | 0.79 |
| Maternal employment (unemployed vs employed*) | -0.18 (-1.50,1.14) | 0.79 | -0.98 (-2.19,0.23) | 0.11 | -0.93 (-2.90,1.04) | 0.35 | 0.08 (-1.33,1.49) | 0.91 |
| Household income level (low vs middle/high*) | -0.34 (-1.22,0.53) | 0.44 | -0.11 (-0.92,0.69) | 0.78 | -1.09 (-2.39,0.22) | 0.10 | -0.51 (-1.45,0.42) | 0.28 |
| Dwelling type (others vs apartments*) | -0.88 (-1.72,-0.04) | 0.04 | 0.05 (-0.72,0.82) | 0.91 | 0.36 (-0.89,1.62) | 0.57 | -0.78 (-1.68,0.11) | 0.09 |
| Father (not cohabiting, cohabiting*) | -0.87 (-1.77,0.02) | 0.06 | -0.62 (-1.44,0.21) | 0.14 | -1.85 (-3.18,-0.51) | 0.007 | -1.08 (-2.04,-0.13) | 0.03 |
| Grandparents (not cohabiting, cohabiting*) | 0.27 (-0.63,1.18) | 0.55 | -0.43 (-1.25,0.40) | 0.31 | -0.46 (-1.81,0.88) | 0.50 | -0.97 (-1.94,-0.01) | 0.048 |
| Female participants (n=94) | Physical domain | | Psychological domain | | Social domain | | Environmental domain | |
|  | Estimate (95%CI) | p value | Estimate (95%CI) | p value | Estimate (95%CI) | p value | Estimate (95%CI) | p value |
| SDQ internalising score | -0.12 (-0.29,0.06) | 0.18 | -0.12 (-0.29,0.05) | 0.16 | -0.21 (-0.41,-0.01) | 0.04 | -0.22 (-0.39,-0.04) | 0.01 |
| SDQ externalising score | -0.09 (-0.22,0.04) | 0.18 | -0.09 (-0.22,0.04) | 0.16 | -0.01 (-0.17,0.15) | 0.89 | -0.14 (-0.27,0) | 0.05 |
| Child age | 0.05 (-0.10,0.21) | 0.51 | -0.03 (-0.18,0.12) | 0.71 | -0.05 (-0.23,0.13) | 0.57 | -0.05 (-0.20,0.11) | 0.55 |
| Maternal age | -0.07 (-0.16,0.02) | 0.11 | -0.01 (-0.10,0.07) | 0.79 | 0 (-0.10,0.10) | 0.99 | 0 (-0.09,0.09) | 0.99 |
| Maternal education levels (middle/low vs high*) | -0.28 (-1.48,0.91) | 0.64 | -0.16 (-1.32,1.00) | 0.79 | 0.22 (-1.18,1.62) | 0.76 | 0.3 (-0.90,1.51) | 0.62 |
| Maternal employment (unemployed vs employed*) | 0.35 (-2.17,2.87) | 0.79 | 0.37 (-2.08,2.82) | 0.77 | -2.74 (-5.70,0.22) | 0.07 | 0.81 (-1.73,3.35) | 0.53 |
| Household income level (low vs middle/high*) | -0.38 (-1.85,1.09) | 0.61 | -0.37 (-1.80,1.06) | 0.61 | -0.22 (-1.95,1.51) | 0.80 | -0.25 (-1.74,1.23) | 0.74 |
| Dwelling type (others vs apartments*) | -0.38 (-1.57,0.80) | 0.52 | -0.47 (-1.63,0.68) | 0.42 | -1.55 (-2.94,-0.16) | 0.03 | -0.9 (-2.09,0.30) | 0.14 |
| Father (not cohabiting, cohabiting*) | -0.55 (-1.87,0.76) | 0.41 | -0.63 (-1.91,0.65) | 0.33 | -2.39 (-3.93,-0.84) | 0.003 | -1.5 (-2.82,-0.17) | 0.03 |
| Grandparents (not cohabiting, cohabiting*) | -1.55 (-2.81,-0.28) | 0.02 | -1.25 (-2.48,-0.02) | 0.05 | -1.77 (-3.26,-0.28) | 0.02 | -1.77 (-3.04,-0.49) | 0.007 |

* Asterisks indicate the reference categories.
